# Active Uptake of Oxycodone at Both the Blood-Cerebrospinal Fluid Barrier and The Blood-Brain Barrier without Sex Differences: A Rat Microdialysis Study

**DOI:** 10.1007/s11095-023-03583-0

**Published:** 2023-08-23

**Authors:** Frida Bällgren, Margareta Hammarlund-Udenaes, Irena Loryan

**Affiliations:** Translational Pharmacokinetics/Pharmacodynamics group (tPKPD), Department of Pharmacy, Uppsala University, Box 580, 75123 Uppsala, Sweden

**Keywords:** blood-brain barrier, blood-cerebrospinal fluid barrier, brain interstitial fluid, cerebrospinal fluid, microdialysis, oxycodone, proton-coupled organic cation (H^+^/OC) antiporter, rat, sex

## Abstract

**Background:**

Oxycodone active uptake across the blood-brain barrier (BBB) is associated with the putative proton-coupled organic cation (H^+^/OC) antiporter system. Yet, the activity of this system at the blood-cerebrospinal fluid barrier (BCSFB) is not fully understood. Additionally, sex differences in systemic pharmacokinetics and pharmacodynamics of oxycodone has been reported, but whether the previous observations involve sex differences in the function of the H^+^/OC antiporter system remain unknown. The objective of this study was, therefore, to investigate the extent of oxycodone transport across the BBB and the BCSFB in female and male Sprague-Dawley rats using microdialysis.

**Methods:**

Microdialysis probes were implanted in the blood and two of the following brain locations: striatum and lateral ventricle or *cisterna magna*. Oxycodone was administered as an intravenous infusion, and dialysate, blood and brain were collected. Unbound partition coefficients (K_p,uu_) were calculated to understand the extent of oxycodone transport across the blood-brain barriers. Non-compartmental analysis was conducted using Phoenix 64 WinNonlin. GraphPad Prism version 9.0.0 was used to perform t-tests, one-way and two-way analysis of variance followed by Tukey’s or Šídák’s multiple comparison tests. Differences were considered significant at p < 0.05.

**Results:**

The extent of transport at the BBB measured in striatum was 4.44 ± 1.02 (K_p,uu,STR_), in the lateral ventricle 3.41 ± 0.74 (K_p,uu,LV_) and in *cisterna magna* 2.68 ± 1.01 (K_p,uu,CM_). These K_p,uu_ values indicate that the extent of oxycodone transport is significantly lower at the BCSFB compared with that at the BBB, but still confirm the presence of active uptake at both blood-brain interfaces. No significant sex differences were observed in neither the extent of oxycodone delivery to the brain, nor in the systemic pharmacokinetics of oxycodone.

**Conclusions:**

The findings clearly show that active uptake is present at both the BCSFB and the BBB. Despite some underestimation of the extent of oxycodone delivery to the brain, CSF may be an acceptable surrogate of brain ISF for oxycodone, and potentially also other drugs actively transported into the brain via the H^+^/OC antiporter system.

**Supplementary Information:**

The online version contains supplementary material available at 10.1007/s11095-023-03583-0.

## Introduction

Several initiatives have been directed towards utilization of active uptake transporters for enhancement of drug CNS exposure [1, 2]. In this regard, the putative proton-coupled organic cation (H^+^/OC) antiporter system (henceforth also referred to as the antiporter system) is a promising target for brain drug delivery due to its association with the phenomenon of active uptake at the blood-brain barrier (BBB) [3–5]. The active uptake, characterized by higher unbound brain interstitial fluid (ISF) concentrations in relation to unbound plasma concentrations, has been documented in rats and was associated with the antiporter system for several marketed drugs such as oxycodone [6, 7], pyrilamine [7, 8], diphenhydramine [9–11], varenicline [12], tramadol [13], memantine [14–16], and bupropion [17]. In spite of almost 20 years of investigation of the phenomenon of active uptake via the H^+^/OC antiporter system, the exact mechanisms of drug transport, also across the blood-cerebrospinal fluid barrier (BCSFB), and its kinetics is still poorly understood. The functional presence of this system in humans remains to be elucidated and is primarily restricted by the inaccessibility to human brain tissue during pharmacokinetic (PK) studies. This implies the use of surrogate matrices of brain ISF such as cerebrospinal fluid (CSF) and blood for assessment of brain exposure in humans. Yet, the dynamic relationship between the brain ISF and CSF concentrations for substrates of the H^+^/OC antiporter system has not been investigated systematically. Hence, understanding of CNS drug disposition of substrates of the H^+^/OC antiporter system and, in particular, CSF exposure, also in relation to its sampling site, is considered one of the critical aspects needed for successful translation from preclinical species to patients and vice versa.

CSF drug exposure is governed by complex interrelated PK processes taking place on blood-brain ISF and blood-choroidal CSF interfaces as well as in the brain parenchyma. A drug from the systemic circulation can reach CSF either directly via passage across the choroid plexus, i.e., the BCSFB, or indirectly by passage across the BBB followed by diffusion and/or convection transport from the brain ISF to CSF in ventricles and *cisterna magna* (CM) entering the CSF circulation [18]. The BBB has the largest surface area for blood-brain exchange and, consequently, has the biggest contribution to brain drug delivery [19–21]. In contrary, the BCSFB has a smaller surface area and its function regarding drug delivery has not been studied as extensively. Given the fact that there are structural and functional differences between the BBB and the BCSFB, also governed by dissimilarities in the molecular composition including transporter expression, the function of the antiporter system at the BCSFB needs investigation [19, 22–24]. To understand the antiporter system’s contribution to drug CNS disposition and, in particular, to CSF exposure, oxycodone, a known substrate of the antiporter system, was used as a model drug in this study [6, 7].

Microdialysis provides unique possibilities to collect data on drug transport longitudinally and from multiple sites. Moreover, microdialysis has been recognized as the most powerful tool for the collection of unbound (free) drug *in vivo* [25–27]. Simultaneous measurements of unbound concentrations in the brain ISF and blood allows assessment of the extent of drug delivery to the brain across BBB by means of the unbound partition coefficient (K_p,uu,brain_) [28, 29]. The blood-brain ISF and blood-choroidal CSF interfaces are in the present paper investigated simultaneously, allowing for a side-by-side comparison of the extent of BBB and BCSFB transport over time, without affecting the CSF hydrodynamics and allowing for a systematic investigation of the relationship between drug concentrations in brain ISF and CSF in different locations, i.e., lateral ventricles (LV) and CM.

Another less investigated factor is the function of the antiporter system in females, as historically, the majority of animal and clinical studies reported are performed in males [30]. Yet, potential sex differences in the function of the antiporter system may impact not only drug PK but also indirectly its pharmacodynamic effect. Studies on oxycodone PK in both sexes are few, and the results are inconsistent [31]. In an early clinical study, lower oxycodone metabolism in women was documented and also attributed to the higher analgesic effect of oxycodone in women compared with that in men [32]. Additionally, oxycodone PK and analgesic effect have been reported to differ between female and male rats [33], and prevailing among female rats in the estrus cycle [34]. Higher clearance in male rats compared to that in females, resulting in a higher systemic exposure in females, was also documented [33]. It is unclear whether there are true discrepancies between females and males in terms of oxycodone CNS exposure and effect. However, differences in the activity of the antiporter system would explain the observed differences in oxycodone CNS exposure and effect. Hence, in the present study, both female and male rats were included to investigate potential sex differences in oxycodone uptake across the brain barriers.

In summary, this study was conducted with the overarching aim to increase the knowledge on the antiporter system’s contribution to drug CNS disposition with focus on CSF exposure, using the model drug, oxycodone. More specifically, the aims were to: 1) Compare the function of the H^+^/OC antiporter system by using the extent of the BBB and the BCSFB transport as a proxy, 2) Study potential sex differences in oxycodone systemic and CNS PK, and 3) Investigate the utility of CSF as a surrogate for brain ISF for assessment of unbound oxycodone concentrations. Hereby, evidence of active uptake of oxycodone, not only across the BBB, but also across the BCSFB is provided. K_p,uu_ values in brain ISF and CSF were sex-independent and on average 46% higher at the BBB than at the BCSFB. Cisternal CSF concentrations underestimated the brain ISF concentrations by approximately 30% and were, thereby, within a drug industry-accepted three-fold range for the prediction of the observed data and indication of equivalence between methods.

## Materials and Methods

### Chemicals

Oxycodone hydrochloride (OxyNorm®, Mundipharma), isoflurane (Isoflo® vet., 100%, solution, Zoetis, Finland), heparin (5000 IU/mL, Heparin LEO), buprenorphine (Temgesic®, 0.3 mg/mL solution for injection, Indivior Eur. Ltd), lidocaine (Xylocain®, 100 mg/mL, cutaneous spray, Aspen Nordic, Denmark) and sterile saline (B. Braun Medical AB, Danderyd, Sweden) were purchased from Distansapoteket Stockholm (Apoteket AB, Stockholm, Sweden). Oxycodone hydrochloride (Eur. Qual D, APL, Kungens Kurva, Sweden) was purchased from Distansapoteket (Falun, Sweden). Oxycodone-D3 and oxycodone-D6 (Cerriliant), sodium chloride, potassium chloride, magnesium chloride, calcium dichloride, ascorbic acid, and potassium dihydrogen phosphate were purchased from Sigma-Aldrich (Saint Louis, USA). Anhydrous dipotassium hydrogen phosphate, formic acid (98–100%) and acetonitrile (gradient grade for liquid chromatography) were purchased from Merck (Darmstadt, Germany). The Milli-Q Academic system (Millipore, Bedford, MA, USA; Resistance 18.2 Ohm; Millipak®Express 20 Filter, 0.22 μm) was purchased from Merck Millipore (Burlington, MA, USA). Dental cement (Dentalon Plus; Heraeus Kulzer GmbH) was purchased from AgnTho’s AB (Lidingö, Sweden). Heparinized saline solution (100 IU heparin/mL) was prepared in house.

### Animals

All experiments were performed on drug-naïve female (n = 8) and male (n = 11) Sprague-Dawley rats (Taconic, Lille Skensved, Denmark) weighing 270–330 g. The rats were group housed by sex at 20–21°C and 45–65% humidity under a 12-hour light-dark cycle with free access to water and food, and acclimatized for one week before experiment. The experiments were in accordance with guidelines from the Swedish National Board for Laboratory Animals and was approved by the Animal Ethics Committee of Uppsala, Sweden (Ethical Approval Dnr. 5.8.18-12230/2019).

The minimal sample size per group required for a two-tailed t-test study was estimated to be four to six, given the probability level (α = 0.05), the anticipated effect size (Cohen’s d = 1.2–1.5), and the desired statistical power level (0.8) [35]. Rats were not randomized, and the study was not blinded.

### Vascular Catheterization and Microdialysis Probe Implantation

Anesthesia was induced by inhalation of 5% isoflurane and maintained by inhalation of 2.5% isoflurane. Additionally, during surgery the inhalation gas was supplemented with 3 L/min oxygen and 0.5 L/min nitrous oxide for sufficient oxygen supply and anesthesia, respectively. The rat was placed on a heating pad and the body temperature was continuously measured via a rectal thermometer (CMA 450 temperature controller; CMA Microdialysis AB, Kista, Sweden). All instruments used during surgery were sterilized at 230–250°C for 15 seconds using a glass bead dry sterilizer (Simon Keller AG, Switzerland). For drug administration, a catheter of polyethylene (PE)-50 tubing fused with a PE-10 tubing connected to silastic tubing was inserted into the left femoral vein (approximately 90% of the rats), or the left jugular vein (approximately 10% of the rats). For blood sampling, a PE-50 tubing fused with a PE-10 tubing was inserted into the left femoral artery. To avoid clotting, the catheters were filled with 100 IU/mL heparinized saline solution. To monitor unbound drug concentrations in plasma, a 10 mm CMA 20 Elite probe (CMA Microdialysis AB, Kista, Sweden) was inserted into the right jugular vein through a guide cannula, and fixed to the pectoral muscles with two sutures. To monitor unbound concentrations of oxycodone in the brain, probes were placed in two of the following locations: striatum (STR), and LV or CM, using a stereotaxic instrument for rats (David Kopf Instruments, Tujunga, CA). A midsagittal incision was made to expose the skull and lidocaine was administered onto the *periosteum* as local anesthesia. In accordance with The Rat Brain in Stereotaxic Coordinates [36], a guide cannula were implanted into STR with the coordinates +0.8 mm anteroposterior and − 2.7 mm lateral to bregma, and − 3.8 mm dorsoventral to the surface of the brain. The LV coordinates were − 0.9 mm anteroposterior, +1.6 mm lateral and − 2.9 mm dorsoventral relative to bregma, and the CM coordinates were − 1.93 mm anteroposterior, +3.15 mm lateral and − 8.1 mm dorsoventral, at an angle of 25° anterior from the dorsoventral axis, 18° lateral from the anteroposterior axis, relative to lambda. After insertion, the guide cannulas were anchored to the skull with anchor screws (flat tip, 2 mm long, 1 mm diameter; AgnTho’s, Lidingö, Sweden) and dental cement. Thereafter, probes were inserted into the guide cannulas. The probes used were: a 3 mm CMA 12 Elite probe for STR and a 1 mm CMA 12 Elite probe for LV and CM (CMA Microdialysis AB, Kista, Sweden). The STR probe was always placed in the right STR. All ends of the cannulas and catheters were put into a plastic cup placed on the posterior surface of the neck out of reach from the rat. The venous and arterial catheters were passed subcutaneously to the neck, and placed into the plastic cup. After surgery, the rat was subcutaneously administered 5 mL/kg isotonic saline solution supplemented with buprenorphine at a dose of 0.01 mg/kg to maintain a healthy hydration and provide postoperative pain relief. Thereafter, the rat was placed in a CMA/120 system for freely moving animals (CMA Microdialysis AB, Kista, Sweden) with free access to food and water, and was allowed to recover for one day prior to experiment. The surgeries and experiments were started between 7 and 10 a.m. for all rats.

### Microdialysis and Dosing Regimens

**Fig. 1:**
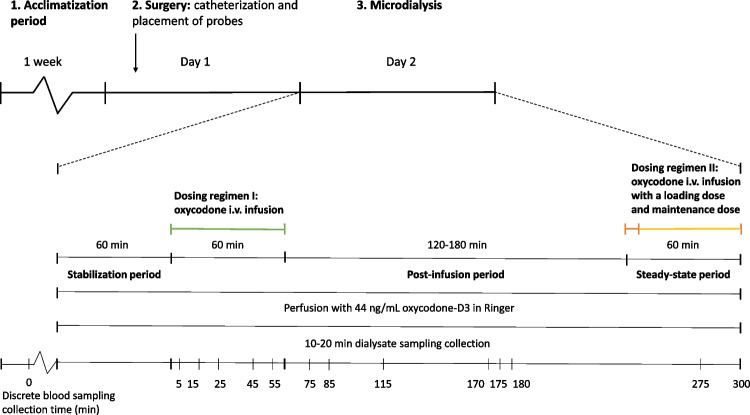
Schematic overview of the experimental design and Dosing regimens. Upon arrival, rats were acclimatized [1] for one week. On Day 1, approximately 24 hours prior the start of experiment, surgery [2] was performed, including catheterization of blood vessels for drug administration and blood sampling, followed by implantation of the CNS and blood probes. The experiment [3] was initiated the next day by a 60 min stabilization period, which implies perfusion of the probes. The rats were administered oxycodone using Dosing regimen I followed by a washout period (n = 14), or Dosing regimen I followed by a washout period and a second administration of oxycodone using Dosing regimen II (n = 5). Throughout the experiment, the probes were perfused with Ringer solution containing the calibrator oxycodone-D3 in 1 μL/min. The dialysate (Ringer solution exiting the probe) was sampled in 10- or 20-min intervals, and blood was sampled at the specified time points. The brain was isolated terminally.

A brief description of the study design is presented in Fig. 1. The microdialysis experiment started approximately 24 h after surgery. The stabilization period lasted for 60 min and included perfusion of the probes with Ringer solution (145 mM NaCl, 0.6 mM KCl, 1.2 mM CaCl_2_, 1 mM MgCl_2_, 0.2 mM ascorbic acid, KH_2_PO_4_ and K_2_HPO_4_; pH 7.4) filtered using a 0.45 μm filter (Acrodisc® syringe filter 0.45 μm GHP membrane; Pall Corporation, Port Washington, NY, USA). The recovery across the probe membrane was determined *in vivo* with retrodialysis by calibrator throughout the experiment [37]. The Ringer solution contained 44 ng/mL of the calibrator oxycodone-D3 and was perfused at a flow rate of 1 μL/min using a CMA 400 Syringe Pump (CMA Microdialysis AB, Kista, Sweden).

#### Dosing Regimen I

Dosing regimen I was performed to investigate the PK parameters in blood and CNS compartments allowing for assessment of the extent of oxycodone transport across the BBB and BSCFB in rats (n_female_ = 8, n_male_ = 11). After the stabilization period, the experiment was divided into two periods: a 60 min constant-rate infusion followed by a 120 min or 180 min wash-out period (Fig. 1). The intravenous (i.v.) infusion rate of oxycodone was 0.3 mg/kg/h administered using a Harvard 22 pump (Harvard Apparatus Inc., Holliston, MA).

#### Dosing Regimen II

In addition to Dosing regimen I, five rats (n_female_ = 3, n_male_ = 2) were also given Dosing regimen II. Dosing regimen II was performed to determine 1) the *in vivo*-assessed intra-brain distribution by estimation of the unbound volume of distribution in the brain (V_u,brain_), 2) the fraction of unbound drug in blood (f_u,blood_), and 3) confirm the K_p,uu_ values to those obtained by Dosing regimen I. After the 180 min post-infusion period in Dosing regimen I, Dosing regimen II was initiated as a combination of an i.v. loading dose of 0.24 mg/kg administered over 2 min, and a maintenance dose of 0.54 mg/kg/h, given as an i.v. infusion over 60 min (Fig. 1). The targeted steady-state concentration was determined based on the maximum unbound concentration in blood obtained by Dosing regimen I.

#### Sampling of Dialysate, Blood and Brain

Throughout the experiment, dialysate samples were collected in pre-weighed polypropylene microvials with polyurethane caps (AgnTho’s, Lidingö, Sweden), in 10- or 20-min intervals. At the end of each dialysate collection interval, the dialysate samples were immediately weighed, capped and stored at 6°C pending bioanalysis.

From each rat, blood samples were collected in heparinized (5 μL 5000 IU/mL heparin) polypropylene Eppendorf tubes (Eppendorf, Hamburg, Germany), before the start of infusion and at 5, 15, 25, 45, 55, 75, 85, 115, and 170 or 175 min, and for Dosing regimen II also at 275 min after the start of infusion. The maximal volume for each blood sample was 180 μL and a maximum of 2 mL blood was sampled from each rat before termination of the experiment. Up to 12 mL of terminal blood was sampled from the heart at 180 or 300 min using vacuum blood collection tubes (BD Vacutainer®, EDTA; Mediq, Utrecht, Netherlands) before isolation of the brain. After sample collection, the blood samples were immediately centrifuged (MicroStar12 centrifuge; VWR International AB, Stockholm, Sweden; or IEC Centra CL2, Labora; IEC international equipment company, Needham Heights, MA, USA) at 10,000 rpm for 5 min, and the plasma was transferred to Eppendorf tubes and stored at −20°C pending bioanalysis.

All brains were isolated and examined to ensure that there was no extensive bleeding around the probes, and to visually confirm the correct positions of the probes. The right STR (location of probe placement) and left STR, as well as the remaining whole brain (without STR; referred to as WB), were collected for bioanalysis of oxycodone to investigate the impact of probe placement on oxycodone total concentrations.

### *In Vivo* Recovery Calculation

To monitor the probe recovery across the probe membranes during the experiment, retrodialysis by calibrator was performed throughout the experiment [37]. The recovery of oxycodone was assumed to be the same as the recovery of the calibrator oxycodone-D3, since the two compounds are almost identical. The probe recovery of the calibrator was calculated as:

1 $$Recovery=\frac{C_{in}-{C}_{out}}{C_{in}}$$

where C_in_ is the average calibrator concentration in the perfusion solution entering the probe, sampled from the Ringer solution containing syringes before and after the experiment. C_out_ is the average concentration of the calibrator in the dialysate samples leaving the CNS and blood probes, collected from each probe throughout the experiment. Average recoveries were calculated for the blood probes (10 mm), STR probes (3 mm) and the LV and CM probes (1 mm), respectively.

### Measurement of Oxycodone Partition into Blood Cells

The partition of oxycodone between blood and plasma (C_b_/C_p_) has previously been studied, and the ratio of oxycodone concentration in blood to that in plasma was 1.3 ± 0.3 [6]. To confirm the partition between blood and plasma in this study, the *in vivo* C_b_/C_p_ was measured in a separate group of rats (n = 3) after administration of an i.v. loading dose of 0.24 mg/kg oxycodone over 2 min, followed by an i.v. maintenance dose of 0.54 mg/kg/h oxycodone, for 240 min. Blood was sampled from the heart by Vacutainer® tubes before decapitation. Blood was centrifuged at 10,000 rpm for 5 min. Thereafter, 25 μL of plasma and 25 μL of blood cells (BC) were individually transferred to new Eppendorf tubes. Samples were stored at 6°C until bioanalysis the following day.

To estimate the hematocrit, blood was collected in heparinized microhematocrit capillary tubes (VWR International, Radnor, USA). The capillary tubes were centrifuged for 5 min (Adams Readacrit®, micro-hematocrit centrifuge model CT-3400). The results were read immediately after centrifugation, by measurement of the length (cm) of the BC volume in the capillary and the full length of the blood volume in the capillary. The hematocrit (Ht) was calculated as follows:

2 $$Ht\ \left(\%\right)=\frac{Capillary\ length\ of\ BC}{Capillary\ length\ of\ plasma\ and\ BC}$$

The partition to blood cells (C_b_/C_p_) was calculated as follows [38]:

3 $$\frac{C_b}{C_p}=1- Ht+ Ht\times \left(\frac{C_{BC}}{C_p}\right)$$

where C_BC_ and C_p_ are the oxycodone concentration in BC and plasma, respectively, and Ht is the rat hematocrit.

### Measurement of *In Vitro* Plasma Protein Binding and Brain Tissue Binding of Oxycodone

The equilibrium dialysis technique was performed to assess the fraction of unbound oxycodone in plasma (f_u,plasma_) and whole brain as well as striatal homogenates (f_u,brain_) *in vitro*, as previously described [39–41]. Briefly, undiluted plasma or brain homogenate (1:9, w:v, diluted in phosphate-buffered saline, PBS, pH 7.4) from drug naïve rats was spiked with oxycodone at concentrations of 30 ng/mL and 300 ng/mL, respectively. To perform the equilibrium dialysis, a Teflon 96-well plate with a semipermeable membrane was used (molecular weight cut off: 12–14 kDa; Model HTD96b, HTDialysis, Gales Ferry, CT, USA). The spiked plasma or brain homogenate was dialyzed against equal volumes of PBS for 6 hours at 37°C with orbital shaking at 200 rpm in MaxQ4450 incubator (Thermo Fisher Scientific, NinoLab, Sweden). Terminally, plasma or brain homogenate, and PBS were sampled and stored at −20°C pending bioanalysis. Matrix matching was applied by adding PBS to the plasma and brain tissue homogenate samples, and vice versa adding plasma or brain tissue homogenate to the PBS samples. Samples were stored at −20°C pending bioanalysis. The ratio of drug concentration in buffer (C_u,buffer_) to that in plasma (C_p_) was calculated to estimate f_u,plasma_:

4 $${f}_{u, plasma}=\frac{C_{u, buffer}}{C_p}$$

The ratio of drug concentration in buffer to that in brain was calculated to estimate f_u,brain_:

5 $${f}_{u, brain}=\frac{\frac{1}{D}}{\left(\left(\frac{1}{f_{u,D}}\right)-1\right)+\frac{1}{D}}$$

where D is the dilution factor and f_u,D_ is the fraction of unbound drug in the diluted brain tissue calculated as:

6 $${f}_{u,D}=\frac{C_{u, buffer}}{C_{tot, tissue}}$$

where C_u,buffer_ and C_tot,tissue_ are the drug concentrations in the buffer and the diluted brain tissue homogenate, respectively. f_u,brain_, measured using the *in vitro* equilibrium dialysis technique, describes non-specific and specific oxycodone binding to brain cellular components.

### Measurement of *In Vitro* Intra-Brain Distribution of Oxycodone

The brain slice method was performed to assess the *in vitro* relationship between total drug amount in the brainand the unbound brain ISF concentration (V_u,brain_, mL × g brain^−1^) in whole coronal brain slices and in the striatal area, as previously described [42–44]. Briefly, six 300 μm slices were obtained from the brain of drug naïve rats (n = 3 per group) using a Leica VT1200 microtome slicer (Leica Microsystems AB, Sweden). The slices were then incubated in 15 mL of artificial extracellular fluid (aECF, pH 7.6 at room temperature) containing 30 ng/mL oxycodone. To match unbound ISF concentration measured *in vivo* in the present microdialysis study, an additional group was included with oxycodone aECF concentration of 300 ng/mL (n = 3 rats). The beaker was covered with a custom fabricated lid fitted with a Teflon fluorinated ethylene-propylene film (Teflon FEP film 50 Å, 12.7 μm). The incubation was performed under 75–80 mL/min oxygen at 45 rpm and 37°C for 5 hours (MaxQ4450 Thermo Fisher Scientific, NinoLab, Sweden). After incubation, aECF was sampled and the slices were cut into two halves, with one half undergoing micro-dissection to obtain STR. Consequently, the intact half of the brain slice (called collectively whole brain, WB) as well as the micro-dissected striatal area from the contralateral half were dried on a filter paper, weighed and homogenized individually in 9 volumes of aECF (w/v) with an ultrasonic processor (VCX-130; Sonics, Chemical Instruments AB, Sweden). The matrices were normalized for bioanalysis by adding blank aECF to the brain slices, and blank brain homogenate (1:4, w:v) to aECF containing samples. Samples were stored at −20°C pending bioanalysis. The *in vitro* V_u,brain_, was calculated as follows:

7 $${V}_{u, brain}=\frac{A_{brain}-{V}_i\times {C}_{u, buffer}}{C_{u, buffer}\times \left(1-{V}_i\right)}$$

where A_brain_ is the amount of the drug in the brain slice, C_u,buffer_ is the measured aECF concentration of the drug and V_i_ is the volume of the remaining aECF layer around the brain slices, here estimated to 0.133 mL × g brain^−1^ using [^14^C]sucrose.

### Bioanalysis

Quantitative analysis of oxycodone, oxycodone-D3 (only in dialysate samples), and oxycodone-D6 as internal standard (IS), in dialysate, plasma, BC and brain samples, respective blanks, as well as standards and QCs prepared in the respective matrices, was achieved by ultraperformance liquid chromatography-tandem mass spectrometry (UPLC-MS/MS). The calibration curves included standards of 0.5, 1, 5, 10, 50, 100 and 150 ng/mL, and QCs of 2, 25 and 75 ng/mL for all matrices.

#### Sample Preparation

Dialysate samples (approximately 9 μL, estimated by the weight), and 8 μL of each of the following: standards (0.5–150 ng/mL), QCs (2, 25 and 75 ng/mL) and blank Ringer solution, were diluted with 25 μL of MilliQ water spiked with an IS concentration of 2–10 ng/mL. Before placing the samples on a 96-well plate, the samples were vortexed (Vortex-Genie® 2; Scientific Industries Inc., Bohemia, NY, USA) for 2 min and spun-down at 10,000 rpm for 15–45 s (ScanSpeed mini centrifuge; LaboGene, Lillerød, Denmark).

Plasma samples from both the microdialysis and the equilibrium dialysis studies were prepared as previously described [45]. Briefly, samples, standards (0.5–150 ng/mL), QCs (2, 25 and 75 ng/mL) and blank plasma were thawed and vortexed for 2 min. A volume of 25 μL of sample was precipitated in 50 μL acetonitrile spiked with an IS concentration of 2–10 ng/mL and vortexed for 2 min, and then centrifuged at 10,000 rpm for 5 min (ScanSpeed mini centrifuge; LaboGene, Lillerød, Denmark). A supernatant volume of 40 μL was transferred to a new microvial and diluted with 80 μL of MilliQ water. The vials were then vortexed for 2 min and put on a 96-well plate. BC samples were included in the plasma sample analysis.

The brain samples were thawed, weighed and homogenized in MilliQ water (1:4, w:v) using an ultrasonicator (Sonics vibra cell, Chemical instruments AB; Sonic materials Inc., Newtown, USA). Brain samples from the microdialysis and brain slice and the equilibrium dialysis studies were treated using the same procedure as described above for plasma samples. The sample volume injected onto the column was 5 μL.

#### Quantification of Oxycodone and Oxycodone-D3

The system for chemical analysis consisted of an Acquity Ultra-Performance Liquid Chromatography instrument, coupled to a Xevo TQ-S Micro mass spectrometer (Waters Corporation, Milford, Massachusetts, USA). The software used for quantification of analytes was MassLynx version 4.2, and TargetLynx (Waters Corporation, Milford, Massachusetts, USA). Chromatographic separation of analytes was performed on an AQUITY UPLC BEH C18-column (1.7 μm, 2.1 × 50 mm) coupled with a VanGuard Pre-Column made of the same material (Waters Corporation, Milford, Massachusetts, USA). The column was maintained at 40°C for optimal peak shape. The sample manager temperature was kept at 5°C. An elution gradient was used for an optimal separation with an initial gradient of 5% Mobile phase B (MPB) and 95% Mobile phase A (MPA). MPB was increased to 45% within 2.5 minutes, and maintained until 3.5 min. Thereafter, MPB was increased to 90% until 4 min, and reversed to 5% until 4.5 min and then maintained until 4.8 min. MPA consisted of 0.1% formic acid in MilliQ water, and MPB consisted of 0.1% formic acid in acetonitrile. The mobile phases were chosen based on low background and high sensitivity, and the elution gradient was chosen based on a low background, reasonable retention time and negligible carry-over. The UPLC retention times of oxycodone were 2.26 ± 0.034, 2.24 ± 0.029 and 2.20 ± 0.0062 min in dialysate, plasma and brain homogenate, respectively. A constant flow rate of 0.3 mL/min was used. To minimize the salt exposure on the MS/MS, the flow from the UPLC was led to waste for the first 1.8 min and after 2.5 min following sample injection. The ionization source was an Electro Spray Ionization probe in positive mode. Quantification was performed using multiple reaction monitoring mode to monitor Parent → Daughter ion (m/z) transitions. The transition modes for oxycodone, oxycodone-D3 and oxycodone-D6 were 316.11 → 298.1 *m/z*, 319.11 → 301.1 *m/z*, and 322.18 → 304.1 *m/z*, respectively. The source dependent parameters maintained for analytes were as follows: capillary voltage 0.55 kV, cone voltage 10 V, source temperature 150°C, desolvation temperature 600°C, desolvation gas (nitrogen) flow 1000 L/h, cone gas (argon) flow 10 L/h, vacuum below 1.3e^−5^ mbar, collision cell pressure below 4e^−3^ mbar, and collision energy 18 V for all compounds.

The calibration curves were constructed using linear regression and a weighing function of 1/X^2^, which resulted in an even residual distribution and similar importance for all the concentrations in the calibration range. The coefficient of determination (R^2^) was ≥0.99. The lower limit of quantification of oxycodone and oxycodone-D3 was set to be equal to the lowest level of the calibration curve, i.e., 0.5 ng/mL, in all matrices. Respective blank matrix samples with and without IS were included in the analytical runs to confirm the absence of contamination and carry-over.

### Data Analysis for PK Parameters

To estimate the unbound oxycodone concentration in blood, STR, LV and CM from the measured microdialysis concentrations, the measured concentration was divided by the recovery of the probe, according to the following equation:

8 $${C}_u=\frac{C_{dialysate}}{Recovery}$$

where C_u_ is the unbound drug concentration at the probe location *in vivo*, C_dialysate_ is the concentration in the dialysate sample, and recovery is the calibrator recovery across the specific probe membrane (Eq. 1). The average probe recoveries for the blood probe (10 mm), STR probe (3 mm), and LV and CM probe (1 mm) were 60.6 ± 16.0% (n = 22), 10.4 ± 4.4% (n = 12), and 3.38 ± 1.21% (n = 3), respectively. The recoveries were relatively stable over time and across dosing regimens, although with some time-independent fluctuations (Table S1).

To estimate total oxycodone concentrations in blood (C_blood_) from the measured total concentrations in plasma, the total concentration in plasma (C_p_) was multiplied with the C_b_/C_p_ value, according to the following equation:

9 $${C}_{blood}={C}_p\times \frac{C_b}{C_p}$$

PK parameters were estimated by non-compartmental analysis using Phoenix 64 WinNonlin (Certara, New Jersey, USA) [46], and denominated as in the software, i.e., the AUC from time 0–180 min (AUC_last_) and that extrapolated to infinity (AUC_inf_obs_), clearance (CL__obs_), volumes of distribution (V_z_obs_ and V_ss_obs_) and terminal half-life (t_1/2_) of unbound oxycodone in blood, STR, LV and CM. Parameters estimated based on the last observed concentration were indicated with _obs and parameters estimated for steady-state were indicated with ss. AUC_inf_obs_ and AUC_last_ were estimated using the linear log trapezoidal method from the time of dosing extrapolated to infinity (inf) based on the last observed concentration (175–235 min) or from time 0 to the last sampled time-point (last), and the first-order rate constant associated with the terminal part of the log-linear curve. The rate constant (Lambda_z) was estimated by linear regression of log concentration between 75 and 240 min. Mean unbound concentrations at steady-state in blood, STR, LV and CM were calculated from samples collected 35 to 55 min after the initiation of steady-state (Dosing regimen II). Mean total blood concentrations at steady-state were calculated from at least two samples collected minimum 35 min after initiation of steady-state. Steady-state clearances (CL_ss_) was estimated based on the rate of infusion (R_0_) and mean steady-state concentrations (C_ss_):

10 $${CL}_{ss}=\frac{R_0}{C_{ss}}$$

Fraction of unbound drug in blood was calculated as follows:

11 $${f}_{u, blood}=\frac{C_{u, blood, ss}}{C_{tot, blood, ss}}$$

where C_u,blood,ss_ is the mean unbound concentration in blood at steady-state and C_tot,blood,ss_ is the mean total concentration in blood at steady-state.

The unbound partition coefficients, describing the extent of drug delivery to striatum (K_p,uu,STR_), lateral ventricle (K_p,uu,LV_) and *cisterna magna* (K_p,uu,CM_) were estimated as follows:

12 $${K}_{p, uu}=\frac{AUC_{u, brain}}{AUC_{u, blood}}$$

and

13 $${K}_{p, uu}=\frac{C_{u, brain, ss}}{C_{u, blood, ss}}$$

where AUC_u,brain_ is the AUC of the unbound drug concentrations in striatal ISF, or lateral ventricular or cisternal CSF, AUC_u,blood_ is the AUC of the unbound drug concentrations in blood, C_u,brain,ss_ is the mean unbound concentration in striatal brain ISF or lateral ventricular or cisternal CSF at steady-state, and C_u,blood,ss_ is the mean unbound concentration in blood at steady-state, measured at 35, 45 and 55 min after the initiation of the loading dose.

To compare the extent of transport between two CNS sites (site 1 and 2), the relative extent was determined based on the ratio of the respective K_p,uu_ value:

14 $${Relative\ extent}_{CNS1- CNS2}=\frac{K_{p, uu, CNS1}}{K_{p, uu, CNS2}}$$

The partition coefficient between total brain concentrations and total blood concentration, K_p,brain_, was estimated as follows:

15 $${K}_{p, brain}=\frac{C_{tot, brain, ss}}{C_{tot, blood, ss}}$$

where C_tot,brain,ss_ and C_tot,blood,ss_ are the total concentration in brain (WB, right- and left STR) and the total concentration in blood at steady-state, respectively.

The *in vivo* apparent unbound volume of distribution in brain (V_u,brain_), describing intra-brain distribution, was calculated as follows:

16 $${V}_{u, brain}=\frac{A_{brain}}{C_{u, brain ISF}}$$

where A_brain_ is the amount of drug in the brain tissue corrected for dilutions during analysis and residual blood in the brain (Eq. 17), and C_u,brainISF_ is the unbound concentration in brain ISF. A_brain_ was calculated, according to Fridén *et al.* [47] as follows:

17 $${A}_{brain}=\frac{C_{brain}-\left({V}_{eff}\times {C}_p\right)-\left({V}_{er}\times {C}_{er}\right)}{1-{V}_{water}-{V}_{er}}$$

where C_brain_, C_p_ and C_er_ are the total drug concentrations in brain, plasma and erythrocytes, respectively. V_er_ and V_water_ are the erythrocyte volume and the apparent plasma water space in brain, respectively. A V_er_ value of 2.13 μL × g brain^−1^ and a V_water_ value of 10.3 μL × g brain^−1^ were used [47]. V_eff_ is the effective plasma space of the drug in the brain, calculated as follows:

18 $${V}_{eff}={f}_{u, plasma}\times {V}_{water}+\left(1-{f}_{u, plasma}\right)\times {V}_{protein}$$

where f_u,plasma_ is the fraction of unbound drug in plasma, V_water_ is the apparent plasma water space and V_protein_ is the apparent vascular space of plasma proteins. The f_u,plasma_ value used was 0.89, and the V_protein_ value used was 7.99 μL × g brain^−1^, which was previously estimated using ^14^C-dextran with a molecular weight of 70 kDa [47].

Conclusions regarding intra-brain distribution were drawn based on the V_u,brain_ value and physiological volumes in brain tissue, where a V_u,brain_ value around 0.2 mL × g brain^−1^ indicates restricted drug distribution in the brain ISF, and a V_u,brain_ value above 1.0 mL × g brain^−1^ indicates brain tissue binding, active uptake into cells and/or distribution to subcellular organelles. The higher the V_u,brain_ value, the more extensive brain tissue binding and/or intracellular distribution [48]. V_u,brain_ values measured *in vivo* were compared with *in vitro* intra-brain distribution parameters obtained in the brain slice (V_u,brain_) and the brain homogenate (f_u,brain_) assays. Inherent inverse correlation between the parameters, i.e., V_u,brain_ ≈ 1/f_u,brain_ was also applied for comparison [49].

### Inclusion and Exclusion Criteria

In all stages of the study, rats were included if they were considered healthy based on Uppsala University assessment guidelines for pain and distress in experimental animals. Rats were placed for acclimatization when the body weight was within the specified range of 270–330 g. Thereafter rats were included in the experiment if catheterization of blood vessels and placements of probes were successful during the surgery (post-mortem verification of correct placement of the CNS probes was determined by visual examination of the brain). Bioanalytical runs were accepted if the precision was within 15% and the accuracy within 15%, except at the lower limit of quantification (LLOQ) (0.5 ng/mL) where an accuracy of ±20% was accepted. The accepted IS recovery of standards and quality control (QC) samples were 80 to 120%. The linearity of the calibration curve was considered acceptable when the coefficient of determination (R^2^) was above 0.99. The AUC_inf_obs_ was used for calculations of K_p,uu_, except in one case where AUC_last_ was used as the extrapolated area (from last time point to infinity) was above 20% of the total estimated area [50].

### Statistical Analysis

Statistical analysis was performed using GraphPad Prism version 9.0.0 for Windows (GraphPad Software, San Diego, California USA, www.graphpad.com). Normal Gaussian distribution of the data was confirmed using Shapiro-Wilk normality test. For comparisons of AUCs, CL, V, t_1/2_ and V_u,brain_ between female and male rats, and between *in vitro* f_u,brain_ in STR and WB, two tailed unpaired t-test was used. For comparison of K_p,uu_ in STR, LV and CM, as well as of t_1/2_ in blood, STR, LV and CM, one-way analysis of variance (ANOVA) followed by Tukey’s multiple comparison test was used. K_p,uu_ comparisons between female and male rats, as well as K_p,uu_ obtained by AUC_inf_obs_ and C_ss_, were performed using two-way ANOVA followed by Šídák’s multiple comparison test. Paired one-way ANOVA followed by Tukey’s multiple comparison test was used to compare total brain concentrations in right STR (location of probe placement), left STR, and whole brain. As ANOVA requires complete data without missing values, data sets with missing values were analyzed by fitting a mixed effects model. The determination coefficients (R^2^) of the ratios of unbound concentration in striatum and lateral ventricle to that in blood (C_u,STR_/C_u,blood_ and C_u,STR_/C_u,blood_) and time, and the ratios and concentration, were obtained by computing a two-tailed correlation. Data are presented as mean ± SD, unless other is specified. Differences were considered significant at p < 0.05.

## Results

### Active Uptake of Oxycodone at the BBB and the BCSFB

**Fig. 2:**
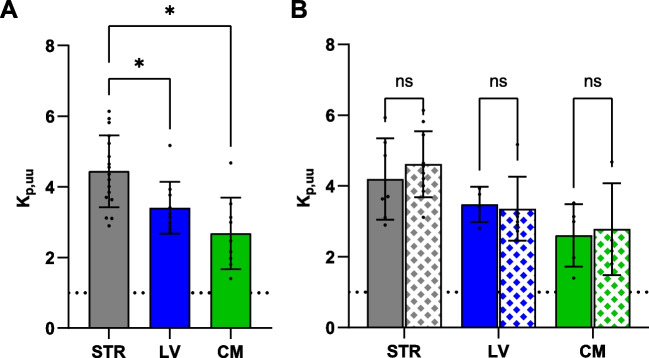
Scatter dot plots of K_p,uu_ estimates. Unbound oxycodone partition coefficient (K_p,uu_) estimates in striatum (STR, gray, N_female_ = 7, N_male_ = 10), lateral ventricle (LV, blue, N_female_ = 4, N_male_ = 6) and *cisterna magna* (CM, green, N_female_ = 5, N_male_ = 4) in (**A**) rats of both sexes, and (**B**) female (filled) and male (pattern) rats obtained by AUC (Eq. 12). The mean is indicated by the top of the bar and the whiskers are representing the SD. Data and detailed statistics are presented in Table I and Table S2-S3. *p < 0.05, ns p > 0.05.

Active uptake of oxycodone was present also at the BCSFB, indicating that the same transporters are present both at the BBB and at the BCSFB. This was shown by higher unbound concentrations in both LV and CM than those in blood, with K_p,uu,LV_ of 3.40 and K_p,uu,CM_ of 2.68, while K_p,uu,STR_ was 4.44 (Eq. 12, Table I and Fig. 2A). The CSF exposure was independent of site of sampling as there were no difference between LV and CM (p = 0.23, Table S2).

**Table I:** K_p,uu_ in Striatum, Lateral Ventricle and *Cisterna Magna* Estimated in Female and Male Rats

Dosing regimen I				Dosing regimen II
	*Both sexes*	*Female*	*Male*	*Both sexes*
K_p,uu,STR_	4.44 ± 1.02 (n = 17)	4.19 ± 1.15 (n = 7)	4.61 ± 0.94 (n = 10)	4.36 ± 0.86 (n = 4)
K_p,uu,LV_	3.41 ± 0.74 (n = 10)	3.48 ± 0.50 (n = 4)	3.36 ± 0.90 (n = 6)	2.92 ± 0.34 (n = 3)
K_p,uu,CM_	2.68 ± 1.01 (n = 9)	2.60 ± 0.88 (n = 5)	2.78 ± 1.30 (n = 4)	2.91 (n = 1)

K_p,uu_ estimated using Eq. 12 for Dosing regimen I, and Eq. 13 for Dosing regimen II. Mean ± SD. Statistical comparisons are presented in Table S2 and S3

The higher exposure in striatal ISF compared to CSF gives a relative extent of oxycodone delivery to STR compared with that to LV of 1.46 ± 0.3, showing that the extent across the BBB was 46% higher compared to that across the BCSFB at the LV site (p = 0.036, n = 9, Fig. 2A, Table S2). The relative extent of oxycodone delivery to STR to that in CM was 1.73 ± 1.01 suggesting 73% higher exposure in STR compared to that in CM (p = 0.017, n = 8, Fig. 2A, Table S2).

No sex differences were observed in the uptake across the barriers in female and male rats (Fig. 2B, Table I, Table S3). As expected, the estimations of partition coefficients were independent of the method of assessment, i.e., by AUC or C_ss_ (Eqs. 12–13, Table I, Table S4).

In addition to differences in exposure between the LV and STR, the t_1/2_ in LV of 45.3 min was approximately 30% longer than those in blood and STR, estimated to 34.7 min (p = 0.0036) and 36.8 min (p = 0.0029), respectively. There were no significant differences in the t_1/2_ in CM estimated to 50.1 min compared to those in blood, STR and LV, due to a high variability (Table S5).

### No Sex Differences in Systemic Blood Pharmacokinetics of Oxycodone

**Fig. 3:**
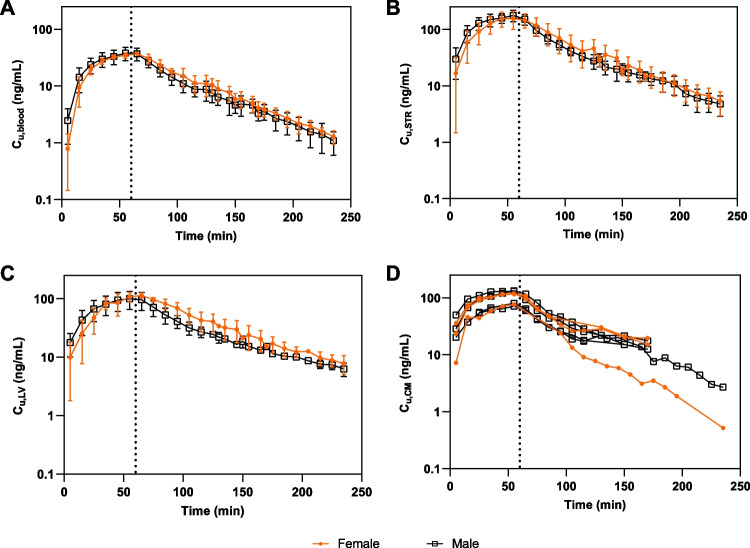
Concentration-time profiles of unbound oxycodone in females and males after administration of Dosing regimen I. Semilogarithmic concentration-time profiles in (**A**) blood (N_female_ = 6, N_male_ = 10), (**B**) striatum (STR) (N_female_ = 6, N_male_ = 10), (**C**) lateral ventricle (LV) (N_female_ = 3, N_male_ = 6), and (**D**) cisterna magna (CM) (N_female_ = 3, N_male_ = 4). Data (**A**-**C**) are presented as mean ± SD or as individual data points (**D**). The dotted line at 60 min represents the stop of infusion.

We did not find any sex differences in the systemic blood PK of oxycodone. The unbound blood concentration-time profiles were similar in female and male rats. Thus, there were no sex differences in clearance or volume of distribution, resulting in no difference in terminal t_1/2_ estimations (Fig. 3A and Table II). Generally, the inter-individual variability measured as coefficient of variation (CV %) was similar between females and males. For instance, CV of K_p,uu,STR_ was 27% in females and 20% in males, and CV of AUC_inf_obs_ was 12% in females and 27% in males (Table S6).

**Table II:** Pharmacokinetic Parameters of Unbound Oxycodone in Blood

Parameter	Unit	Both sexes	Female	Male
*Dosing regimen I*				
Area under the unbound concentration-time curve, AUC_inf_obs_	*min×ng×mL*^*−1*^	3080 ± 670 (n = 16)	3170 ± 370 (n = 6)	3020 ± 810 (n = 10)
AUC from time 0–180 min, AUC_last_	*min×ng×mL*^*−1*^	2890 ± 620 (n = 16)	2960 ± 330 (n = 6)	2850 ± 760 (n = 10)
Clearance, CL__obs_	*mL×min*^*−1*^ *× kg*^*−1*^	101 ± 19 (n = 16)	95.6 ± 10.5 (n = 6)	105 ± 23 (n = 10)
Terminal volume of distribution, V_z_obs_	*mL×kg*^*−1*^	5040 ± 820 (n = 16)	4930 ± 810 (n = 6)	5110 ± 860 (n = 10)
Volume of distribution at steady-state, V_ss_obs_	*mL×kg*^*−1*^	4850 ± 830 (n = 16)	5070 ± 430 (n = 6)	4720 ± 1000 (n = 10)
Terminal half-life, t_1/2_	*min*	34.7 ± 4.0 (n = 18)	35.2 ± 5.3 (n = 8)	34.3 ± 2.8 (n = 10)
*Dosing regimen II*				
C_u,blood,ss_	*ng×mL*^*−1*^	69.0 ± 10.5 (n = 4)	NA	NA
C_tot,blood,ss_	*ng×mL*^*−1*^	132 ± 7 (n = 3)	NA	NA
CL_ss_	*mL×min*^*−1*^ *× kg*^*−1*^	133 ± 20 (n = 4)	NA	NA
C_b_/C_p_	*unitless*	1.21 ± 0.08 (N = 3, n = 6)	NA	NA
f_u,blood_	*unitless*	0.66 ± 0.34 (n = 4)	NA	NA

Mean ± SD. Comparisons between female and male rats were performed using two-tailed unpaired t-tests with the following results: AUC_inf_obs_ (p = 0.67), AUC_last_ (p = 0.74), CL__obs_ (p = 0.38), V_z_obs_ (p = 0.69), V_ss_obs_ (p = 0.44) and t_1/2_ (p = 0.63)

Oxycodone partition between blood and plasma (C_b_/C_p_, Eq. 3) was estimated *ex vivo* to 1.21 ± 0.08 (N = 3, n = 6) based on a rat hematocrit estimation of 46.3 ± 3.8% (N = 3, n = 6, Eq. 2). The fraction of unbound oxycodone in blood (f_u,blood_) was estimated *in vivo* to 0.66 ± 0.34 (n = 4) using mean steady-state concentrations obtained after Dosing regimen II (Eq. 11). The fraction of unbound oxycodone in plasma (f_u,plasma_) was estimated *in vitro* to 0.87 ± 0.03 (N = 3, n = 3).

### Rapid Equilibrium of Oxycodone Uptake to Striatum, Lateral Ventricle and *Cisterna Magna*

**Fig. 4:**
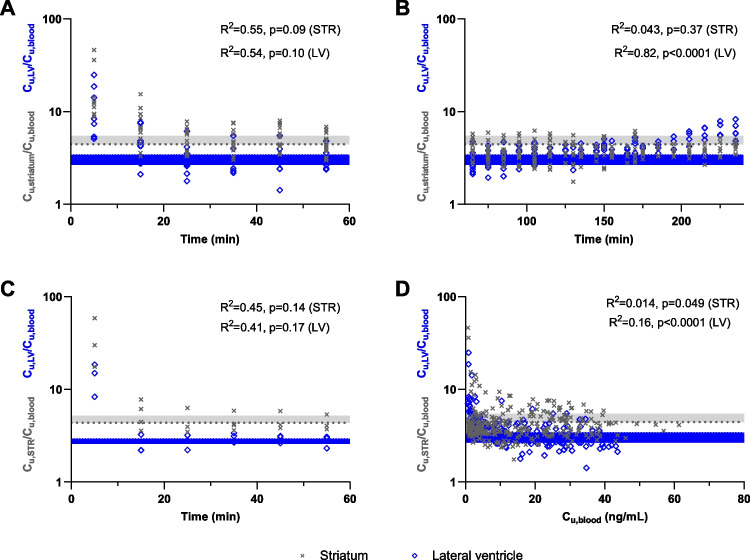
Unbound concentration ratios in striatum and lateral ventricle *versus* time and unbound blood concentration. The relationships of the single time point ratios of the unbound oxycodone concentration in striatum and lateral ventricle to that in blood (C_u,STR_/C_u,blood_, gray cross; C_u,LV_/C_u,blood_, blue empty diamonds), and (**A**) time during infusion (Dosing regimen I, N_STR_ = 17, N_LV_ = 10), (**B**) time after infusion (Dosing regimen I), (**C**) time during infusion (Dosing regimen II, N_STR_ = 4, N_LV_ = 3) and (**D**) unbound blood concentration (Dosing regimen I). The horizontal dotted lines represent mean K_p,uu_ in STR (gray) and LV (blue) obtained by Dosing regimen I (**A**, **B**, **D**) and Dosing regimen II (**C**), and the shadowed areas represent the respective SD. The goodness of fit is described by the determination coefficients (R^2^) which were obtained by computing a two-tailed correlation.

There was a very rapid equilibration of oxycodone uptake to all three sites of the brain. The unbound concentration ratios across the BBB and the BCSFB (C_u,STR_/C_u,blood_ and C_u,LV_/C_u,blood_, respectively) were initially over 10 times higher than during later time-points, yet, the ratios quickly stabilized (Fig. 4A-C, Eqs. 12–13). A similar pattern was observed in CM. Already at the second sampling period, the values approached mean K_p,uu_ values. There was a tendency to a time-dependent increase in unbound oxycodone concentration in LV after the end of infusion, but not in STR (R^2^ = 0.82, p < 0.0001, and R^2^ = 0.043, p = 0.37, in LV and STR, respectively). There is, however, no relationship between the ratios and the unbound concentration in blood (R^2^ = 0.014, p = 0.049 in STR, and R^2^ = 0.16, p < 0.0001 in LV, Fig. 4D).

### Intra-Brain Distribution of Oxycodone

The intra-brain distribution of oxycodone showed no site-specific differences in total concentrations or total brain-to-blood concentration ratio, K_p_ between left and right STR, indicating no influence of the microdialysis probe placement on the distribution and binding of oxycodone (Table S7). Individual total concentrations and the individual unbound striatal ISF concentrations measured with microdialysis in right STR resulted in an average V_u,brain_ of 2.11 ± 0.95 mg × g brain^−1^ (n = 4, Eq. 16, Table S7), indicating distribution into brain parenchymal cells. V_u,brain_ estimated in the brain slice assay at 300 ng/mL, matching average unbound striatal ISF concentration at steady-state, was 3.88 ± 0.20 mL × g brain^−1^ (n = 3, Eq. 7) and was 1.8-fold higher than the *in vivo* measured unbound volume of distribution (p = 0.035 for unpaired t test with Welch’s correction). The *in vitro*-estimated V_u,brain_ values at 30 ng/mL were 4.05 ± 0.33 (N = 3, n = 2) and 4.4 ± 0.1 (N = 3, n = 2) in WB and STR, respectively, and were slightly higher compared to V_u,brain_ values obtained at 300 ng/mL. The *in vitro*-estimated fraction of unbound oxycodone in brain (f_u,brain_) was 0.45 ± 0.12 (N = 3, n = 1–2) and 0.34 ± 0.015 (N = 3, n = 1–2) in WB and STR, respectively, without a difference between the two (p > 0.99). The *in vitro*-estimated f_u,brain_ measured for striatum corresponding to a V_u,brain_ of 2.94 mL × g brain^−1^ calculated from the 1/f_u,brain_ relationship.

## Discussion

This study provides novel *in vivo* evidence that the H^+^/OC antiporter system is present and functioning at the BCSFB, based on an oxycodone K_p,uu,LV_ of 3.41, as well as confirming its presence at the BBB [6], based on K_p,uu,STR_ of 4.4. On average, the individual relative extent of oxycodone uptake across the BBB to that across the BCSFB was 46% higher across the BBB compared with that across the BCSFB. Although the extent of uptake is significantly different at the barriers, data suggest that drugs that are actively taken up at the BBB with this system will likely also have higher concentrations in CSF than in blood, yet, CSF concentrations will somewhat underestimate brain ISF concentrations. As the K_p,uu_ ratios are within three-fold, CSF measurements could be used as a surrogate for brain delivery. This is relevant for oxycodone and likely also to other drugs that are actively taken up via the H^+^/OC antiporter system, like diphenhydramine, tramadol, memantine and others.

From the conceptual point of view, it is important to bear in mind that in *in vivo* conditions the assessment of the contributive role of each brain barrier, including BBB and BCSFB, to drug CSF exposure is very challenging. In case of oxycodone, the entry into the CSF could occur via transport across the BCSFB and the BBB followed by drainage from brain ISF. There are several studies showing evidence of ISF bulk flow draining into CSF, and that a small proportion of CSF is recycling into ISF [51–54]. However, the extent of the ISF-CSF exchange is not fully understood from both physiological and PK points of view. In the light of this study, the transport of oxycodone to the CSF from the blood across BCSFB is likely higher than that from the brain ISF as no delay was observed in the oxycodone concentration-time profile for both LV and CM, with parallel profiles across all investigated compartments.

Higher extent of oxycodone in brain ISF than in CSF in both the LV and CM, may indicate a lower expression of the transporter at the BCSFB than at the BBB. In spite of strong evidence from *in vivo* and *in vitro* investigations supporting the active uptake phenomenon [55], the gene(s) coding for the antiporter system has not yet been confirmed. Remarkably, functional involvement of two protein components, transmembrane 7 superfamily member 3 (TM7SF3) and LHFPL tetraspan subfamily member 6 (LHFPL6), has been found in a recent proteomics-based transporter identification study performed in *in vitro* cell lines [5]. The expression levels of the antiporter system proteins at CNS barriers are to our knowledge not known. However, in an early study, *in vivo* and *in vitro* experiments of isolated rabbit choroid plexus indicated carrier-mediated uptake of diphenhydramine at the BCSFB [56]. In mouse brain endothelial cells, the TM7SF3 mRNA expression level was higher than that of breast cancer resistance protein (BCRP), and in human brain endothelial cells, the LHFPL6 mRNA expression level was higher than that of glucose transporter 1 (GLUT1) and L-type amino acid transporter 1 (LAT1) [12].

Observed dissimilarities in absolute concentrations of oxycodone between ISF and CSF are critical for evaluation of CNS exposure in humans as CSF is often used as a surrogate for brain ISF [18, 57]. Given varying conclusions on the predictive ability of CSF for brain ISF of drugs with active transport mechanism [23, 58, 59], it is crucial to examine this relationship. In this study, observed similar CSF exposure in LV and CM suggests that the CSF concentration of oxycodone is independent of the location of sampling between these two. However, CSF is often sampled by lumbar puncture in humans while from CM in research animals [57]. Remarkably, the ratio of oxycodone AUC in lumbar CSF to that in total plasma was reported to be 1.18 in humans [60]. By considering the correction for plasma protein binding (f_u,plasma_ of 0.55 based on FDA label) and neglected drug binding in CSF, it can be concluded that there is an active uptake of oxycodone into the CSF in humans with an estimated K_p,uu,CSF_ of ca 2 which is within the range of K_p,uu,CM_ (mean value of 2.68) observed in rats in this study. Yet, potential differences in concentration of oxycodone between lumbar and CM or LV CSF should be considered for evaluation of the extent of the BCSFB transport in humans.

In our study, initially higher concentration ratios were observed at both the BBB and the BCSFB independent of dosing regimen (Fig. 4A-C). The lack of relationship between oxycodone C_u,STR_/C_u,blood_ and C_u,STR_/C_u,blood_ and unbound blood concentration was confirmed by the low coefficients of determination (Fig. 4D). The rapid brain uptake of oxycodone, with an initial C_u,STR_/C_u,blood_ above 10 followed by a stabilization at 3.03 was previously documented [6], with similar trends also found for diphenhydramine [61] and bupropion [17]. The latter could reflect a phenomenon of saturation of oxycodone transport at higher plasma concentration It was previously suggested that an overshoot in K_p,uu_ may also be observed due to a reduced proton gradient over time [17], as the antiporter system is pH sensitive [62]. However, we do not see any obvious reason to suspect such a rapid shift in pH. Another potential explanation for initially higher single time point concentration ratios is that the initially lower unbound concentrations have larger uncertainties due to allowance of ±20% deviation in the quantification of lower concentrations. If this is the case the uptake should increase again when oxycodone concentrations in blood decrease over time which is not the case at the BBB. Additionally, not all concentration ratios at low blood concentrations are above the average K_p,uu_ values.

As the conclusions of active uptake to the CNS is dependent on a correct estimation of unbound concentrations, the determination of recovery is crucial for correction of the incomplete recovery across the microdialysis probe. To minimize the uncertainties in recoveries and unbound concentration estimations, retrodialysis by calibrator was applied. The recovery was monitored continuously in each individual *in vivo*, which has been suggested as a reliable method to assess probe recovery [37], and the recovery was generally stable over time. The average recoveries of the 10 mm and 3 mm probes were both above 10%, while that of the 1-mm probes was 3.38%, which is considered low. A low recovery is resulting in uncertainties in the unbound concentration estimations. However, in the LV and CM, this short probe length is required. A decreased flow rate could have increased the recovery, however, the frequent 10-min sampling interval applied with 1 μL/min was needed with respect to temporal resolution and the sample volume required for analysis [63].

Our results did not reveal any sex differences in neither oxycodone transport across CNS barriers nor systemic PK, which is contrary to previously reported sex and/or estrus cycle differences presented for oxycodone analgesic effect, brain exposure, clearance, metabolism and oral bioavailability [33, 34]. The analgesic effect of oxycodone was not examined in this study, still, the analgesia has previously been documented to correlate with unbound oxycodone concentrations in brain [34]. Also, estrus cycle was not assessed in this study. However, as the hormonal profile in rodents is rapid with a 4-day cycle [64], and as our experiments were conducted on eight females from different litters and at different time points, it is likely that the results represent the overall cycle. Even though it has been suggested that pooling females in different cycle phases may mask differences dependent on the estrus cycle [64], the observed variability in oxycodone CNS delivery and systemic PK was similar or lower among females than among males, and no outliers were detected. Important differences between our study and previously reported studies are higher oxycodone doses and oral administration, which may contribute to the inconsistent results [33, 34]. Sex differences in analgesia were reported to be present at higher but not at lower oxycodone doses in mice [33]. Also, the oral bioavailability of oxycodone is very low in rats and is subject to sex differences, with females having a 5-fold higher bioavailability [33]. The brain exposure of unbound oxycodone was reported to differ between females in diestrus and male rats, despite similar plasma levels of oxycodone [34]. This may indicate differences in oxycodone brain delivery, however, the sex- and estrus cycle differences in oxycodone CNS exposure and analgesia were attributed by the authors to differences in the brain CYP2D oxycodone metabolism. These observations are difficult to evaluate as oxycodone was administered orally and blood PK was not characterized, and limited to determination of plasma concentration at a single time point. Altogether, the interpretation of previously observed sex differences in oxycodone CNS exposure and analgesia in the light of our own findings is challenging due to multiple confounding factors inherent to the study designs including oxycodone dose, route of administration with low oral bioavailability, and sampling of blood and brain. Yet, our findings from a dedicated PK study with longitudinal sampling of unbound concentrations of oxycodone in the blood and several brain compartments after administration of clinically relevant doses, are ruling out the existence of sex differences in the extent of oxycodone transport across brain barriers in healthy rats.

## Conclusions

This study provides novel *in vivo* evidence of functional presence of the putative H^+^/OC antiporter system at the BCSFB, yet, reaching somewhat lower K_p,uu_ values than that at the BBB for oxycodone. Thus, CSF concentrations slightly underestimated striatal ISF concentrations. Still, CSF could be an acceptable surrogate for brain ISF for drugs handled by this transporter, as the deviation is within three-fold. The lack of sex difference in the active uptake of oxycodone to STR, LV and CM, suggests that the antiporter system likely contributes equally to CNS exposure in female and male rats. A high extent of drug delivery to the brain is beneficial for CNS drug delivery and reduces the risk of peripheral side effects. These findings therefore also widen the possibilities for characterization of the antiporter system as a viable drug delivery target to enable discovery and development of novel CNS drug candidates. Hence, targeting the H^+^/OC antiporter system in CNS drug development is of great promise for brain-specific drug delivery, and the present study indicates that measuring CSF may be a good alternative in species where brain tissue sampling or microdialysis cannot be performed, given that these species express the antiporter system to the same extent as in rats.

### Supplementary Information

ESM 1 (DOCX 78 kb)

## Data Availability

Data are available from the corresponding authors (IL and FB) upon request.

## Abbreviations

A_brain_: Amount of drug in the brain
AUC: Area under the concentration-time curve
AUC_inf_obs_: Area under the concentration-time curve from time 0 extrapolated to infinity
AUC_last_: Area under the concentration-time curve from time 0 to the last time point
AUC_tot_: Area under the total concentration-time curve
AUC_u_: Area under the unbound concentration-time curve
BBB: Blood-brain barrier
BC: Blood cells
BCSFB: Blood-cerebrospinal fluid barrier
C_b_/C_p_: The partition of drug between blood and plasma
C_blood_: Total drug concentration in blood
C_brain_: Total drug concentration in brain
C_tot,blood,ss_: Total drug concentration in blood at steady-state
C_tot,brain,ss_: Total drug concentration in brain at steady-state
C_tot,tissue_: Total drug concentration in diluted brain tissue
C_er_: Total drug concentration in erythrocytes CL__obs_ Drug clearance based on the last observed concentration
CM: Cisterna magna
CNS: Central nervous system
C_p_: Total drug concentration in plasma
CSF: Cerebrospinal fluid
C_ss_: Drug concentration at steady-state
C_tot,brain_: Total drug concentration in brain
C_u_: Unbound drug concentration
f_u,blood_: Unbound fraction of the drug in blood
f_u,brain_: Unbound fraction of the drug in brain.
f_u,plasma_: Unbound fraction of the drug in plasma
Ht: Hematocrit
IS: Internal standard
ISF: Interstitial fluid
i.v.: Intravenous
K_p,brain_: Total brain-to-blood partition coefficient
K_p,uu_: Unbound brain-to-blood partition coefficient
K_p,uu,CM_: Unbound cisternal (cisterna magna) CSF-to-blood partition coefficient
K_p,uu,LV_: Unbound lateral ventricular CSF-to-blood partition coefficient
K_p,uu,STR_: Unbound striatal ISF-to-blood partition coefficient
LAT1: L-type amino acid transporter 1
LHFPL6: LHFPL tetraspan subfamily member 6 protein
LLOQ: Lower limit of quantification
LV: Lateral ventricle
PET: Positron emission tomography
PK: Pharmacokinetics
QC: Quality control
SD: Standard deviation
SS: Steady-state
t_1/2_: Terminal half-life of the drug
TM7SF3: Transmembrane 7 superfamily member 3
UPLC-MS/MS: Ultraperformance liquid chromatography-tandem mass spectrometry
V_eff_: Effective plasma space of the drug
V_er_: Erythrocyte volume in blood
V_protein_: Apparent vascular space of plasma proteins
V_ss_obs_: An estimate of volume of distribution at steady-state based on the last observed concentration, for non-steady-state data
V_u,brain_: Apparent brain volume of distribution of the drug
V_water_: Apparent plasma water space
V_z_obs_: Terminal volume of distribution based on the last observed concentration
