# Supplementary material for: Active Uptake of Oxycodone at Both the Blood-Cerebrospinal Fluid Barrier and The Blood-Brain Barrier without Sex Differences: A Rat Microdialysis Study
Source: Pharm Res. 2023 Aug 23;40(11):2715–30. doi: 10.1007/s11095-023-03583-0 (PMC10733202; doi:10.1007/s11095-023-03583-0)
Supplement: Supplementary file 1 — (DOCX 78 kb) [file 11095_2023_3583_MOESM1_ESM.docx]

## Supplementary material

### Tables

**Table S1. Representative individual probe recoveries (%) for probes in blood (10 mm), striatum (3 mm), and lateral ventricle (1 mm).**

| **Time (min)** | **Blood** | | **STR** | | **LV** | |
| --- | --- | --- | --- | --- | --- | --- |
|  | *Rat 1* | *Rat 2* | *Rat 1* | *Rat 2* | *Rat 1* | *Rat 2* |
| 5 | 64.7 | 53 | 10.8 | 7.7 | 3.07 | 4.55 |
| 15 | 53.3 | 53.7 | 7.7 | 8.2 | 2.69 | 5.62 |
| 25 | 59 | 51.6 | 5.6 | 7.2 | 6.7 | 3.74 |
| 35 | 68.5 | 53.3 | 13 | 7.8 | 1.66 | 4.66 |
| 45 | 64.8 | 55.9 | 12.3 | 7.7 |  | 2.87 |
| 55 | 68.7 | 56.7 | 2.5 | 7.5 |  | 3.95 |
| 65 | 65.4 | 59 | 8.9 | 8 | 3.64 | 5.29 |
| 75 | 68.5 | 56.3 | 18.4 | 8.8 | 2.45 | 5.2 |
| 85 | 72.6 | 51.8 | 8.8 | 8.7 | 4.44 | 5.55 |
| 95 | 68.6 | 52.4 | 22.1 | 7.7 | 1.33 | 5.33 |
| 105 | 73.9 | 52.3 | 9.9 | 6.9 |  | 7.16 |
| 115 | 73.7 | 52.1 | 11.5 | 7.6 | 7.05 | 5.55 |
| 245 |  | 51.4 |  | 8 |  | 3.71 |
| 255 |  | 49.6 |  | 7.4 |  | 4.94 |
| 265 |  | 48.8 |  | 7.6 |  | 4.48 |
| 275 |  | 51.5 |  | 7.5 |  | 4.88 |
| 285 |  | 52.1 |  | 6.9 |  | 3.43 |
| 295 |  | 49 |  | 3 |  |  |
| ***Average (ALL)*** | ***66.81*** | ***52.81*** | ***10.96*** | ***7.46*** | ***3.67*** | ***4.76*** |
| ***SD ALL*** | ***6.01*** | ***2.71*** | ***5.28*** | ***1.22*** | ***2.05*** | ***1.03*** |
| ***CV ALL (%)*** | ***9.00*** | ***5.13*** | ***48.18*** | ***16.39*** | ***55.78*** | ***21.57*** |
| ***Average (DR I)*** | ***66.81*** | ***54.01*** | ***10.96*** | ***7.82*** | ***3.67*** | ***4.96*** |
| ***Average (DR II)*** |  | ***50.40*** |  | ***6.73*** |  | ***4.29*** |

Dosing regimen I (DR I) was administrated from time 0-60 min and dosing regimen II (DR II) from 240-300 min. Cells are left empty where the estimation of recovery was not available. The average probe recoveries for the blood probe (10 mm), STR probe (3 mm), and LV and CM probe (1 mm) were 60.6±16.0% (n=22), 10.4±4.4% (n=12), and 3.38±1.21% (n=3), respectively.

**Table S2. Statistical test details of comparisons of K_p,uu_ by probe location.**

| Test details | STR vs. LV | STR vs. CM | LV vs. CM |
| --- | --- | --- | --- |
| Mean 1 | 4.44 | 4.44 | 3.405 |
| Mean 2 | 3.405 | 2.680 | 2.68 |
| Difference between the means, Mean Diff. | 1.035 | 1.760 | 0.7253 |
| Standard error of the Mean Diff. | 0.337 | 0.465 | 0.436 |
| n1 | 17 | 17 | 10 |
| n2 | 10 | 9 | 9 |
| Ratio reported by the Tukey’s test, q | 4.348 | 5.353 | 2.354 |
| Degrees of freedom, DF | 8 | 7 | 33 |
| 95% confidence interval of the Mean Diff. | 0.07306 to 1.997 | 0.3907 to 3.130 | -0.3438 to 1.794 |
| Adjusted p value | 0.0364 | 0.0165 | 0.2338 |

K_p,uu_ estimated using AUC (Eq. 12). Data are presented in Fig. 2A and Table 1. The tests used were a matched (STR vs. LV and STR vs. CM) and not matched (LV vs. CM) one-way ANOVA followed by a Tukey’s multiple comparison tests.

**Table S3. Statistical test details of comparisons of K_p,uu_ by probe location and sex.**

| Test details  Female – Male | STR | LV | CM |
| --- | --- | --- | --- |
| Predicted (LS) mean 1 | 4.193 | 3.477 | 2.603 |
| Predicted (LS) mean 2 | 4.613 | 3.357 | 2.776 |
| Predicted (LS) mean diff. | -0.4195 | 0.1204 | -0.1734 |
| SE of diff. | 0.4832 | 0.6330 | 0.6578 |
| N1 | 7 | 4 | 5 |
| N2 | 10 | 6 | 4 |
| DF | 30 | 30 | 30 |
| 95% CI of diff. | -1.641 to 0.8023 | -1.480 to 1.721 | -1.837 to 1.490 |
| Adjusted p value | 0.77 | >0.99 | >0.99 |

K_p,uu_ estimated using AUC (Eq. 12). Data are presented in Fig. 2B and Table 1. The test used was a two-way ANOVA and a Šídák’s multiple comparison test. Predicted (LS) mean 1 and 2 are the predicted least squares mean values of K_p,uu_ assessed by the methods. Predicted (LS) mean diff. is the difference between the predicted least squares means. SE of diff. is the standard error of the difference between the two means. N are the number of values. DF is the degrees of freedom. 95% CI of diff. is the 95% confidence interval of the means.

**Table S4. Statistical test details of comparisons of K_p,uu_ obtained by Dosing regimen I (AUC) and Dosing regimen II (**C_ss_**).**

| Test details  AUC – C_ss_ | STR | LV |
| --- | --- | --- |
| Predicted (LS) mean 1 | 4.440 | 3.405 |
| Predicted (LS) mean 2 | 4.363 | 2.921 |
| Predicted (LS) mean diff. | 0.07724 | 0.4838 |
| SE of diff. | 0.5111 | 0.6054 |
| N1 | 17 | 10 |
| N2 | 4 | 3 |
| DF | 38.00 | 38.00 |
| 95% CI of diff. | -1.199 to 1.354 | -1.028 to 1.996 |
| Adjusted p value | 0.9983 | 0.8140 |

K_p,uu_ estimated using AUC (Eq. 12) and C_ss_ (Eq. 13). Data are presented in Table 1. The test used was a two-way ANOVA and a Šídák’s multiple comparison test. Predicted (LS) mean 1 and 2 are the predicted least squares mean values of K_p,uu_ assessed by the methods. Predicted (LS) mean diff. is the difference between the predicted least squares means. SE of diff. is the standard error of the difference between the two means. N are the number of values. DF is the degrees of freedom. 95% CI of diff. is the 95% confidence interval of the means.

**Table S5. Estimations of t_1/2_ of unbound oxycodone in blood, striatum, lateral ventricle and *cisterna magna*.**

|  | Unit | Blood | STR | LV | CM |
| --- | --- | --- | --- | --- | --- |
| Half-life (t_1/2_) | *min* | 34.7±4.0 (n=18) | 36.8±3.8 (n=18) | 45.3±7.0 (n=11)^§,#^ | 50.1±22.6 (n=9) |

Estimated half-life (t_1/2_) of unbound oxycodone after Dosing regimen I. Mean ± SD. Comparisons were performed using matched (Blood vs. STR, Blood vs. LV, blood vs. CM, STR vs. LV, STR vs. CM) and not matched (LV vs. CM) one-way ANOVA and Tukey’s multiple comparison tests: blood vs. STR (p=0.20), ^§^ blood vs. LV (p=0.0036), blood vs. CM (p=0.15), ^#^STR vs. LV (p=0.0029), STR vs. CM (p=0.27), LV vs. CM (p=0.69).

**Table S6. Estimated coefficients of variation (CV, %) of pharmacokinetic parameters in both sexes**.

| Parameter | Both sexes | Female | Male |
| --- | --- | --- | --- |
| K_p,uu,STR_ | 23 (n=17) | 27 (n=7) | 20 (n=10) |
| K_p,uu,LV_ | 22 (n=10) | 14 (n=4) | 27 (n=6) |
| K_p,uu,CM_ | 38 (n=9) | 34 (n=5) | 47 (n=4) |
| AUC_inf_obs_ | 22 (n=16) | 12 (n=6) | 27 (n=10) |
| CL__obs_ | 19 (n=16) | 11 (n=6) | 22 (n=10) |
| V_z_obs_ | 16 (n=16) | 16 (n=6) | 17 (n=10) |
| V_ss_obs_ | 17 (n=16) | 8 (n=6) | 21 (n=10) |

CV (%) of K_p,uu_ (STR, LV and CM), CL and V_ss_ were compared between females and males using an unpaired two-taled t-test (p=0.27).

**Table S7. No significant differences in total brain concentration at steady-state (C_ss,brain_**)**,** **total brain-to-blood partition coefficient (K_p,brain_) and apparent unbound volume of distribution in brain (V_u,brain_) in whole brain, right and left striatum.**

| Parameter/location | Units | Whole brain | Left striatum | Right striatum |
| --- | --- | --- | --- | --- |
| C_ss,brain_ | *ng ×g brain^-1^* | 587.5±243.2 (n=5) | 516.1±157.5 (n=5) | 526.3±185.9 (n=4) |
| K_p,brain_ | *Unitless* | 3.37±0.43 (n=5) | 3.14±0.80 (n=5) | 3.26±0.83 (n=4) |
| V_u,brain_ | *mL×g brain^-1^* | 2.23±1.23  (n=5)* | 1.93±0.88 (n=5)* | 2.11±0.95 (n=4) |

Data presented as mean ± SD and number of biological replicates. Comparisons between whole brain, left striatum and right striatum were performed using matched one-way ANOVA Tukey’s multiple comparison tests of C_SS,brain_ (p=0.22), K_p,brain_ (p=0.87), and V_u,brain_ (p=0.23).* similar unbound concentrations as obtained from the right striatal ISF is assumed.

### Figure legends

**Figure S1. Mean total concentration-time profiles of oxycodone in female and male rats.** The concentration-time profiles of oxycodone in female (orange dots, n=6) and male (black empty squares, n=9) rats after Dosing regimen I. Data are presented as individual data points. The dotted line at 60 minute represents the stop of infusion.

### Figures


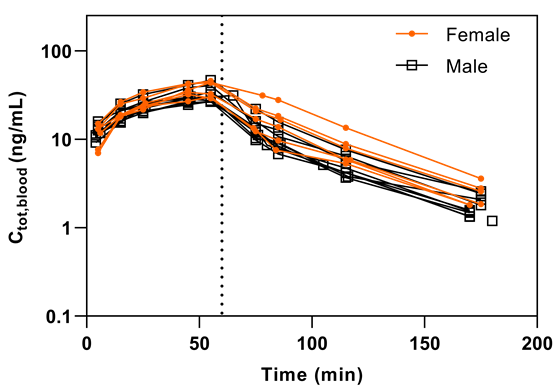


**Figure S1**.
